# A single amino acid residue in bank vole prion protein drives permissiveness to Nor98/atypical scrapie and the emergence of multiple strain variants

**DOI:** 10.1371/journal.ppat.1010646

**Published:** 2022-06-22

**Authors:** Laura Pirisinu, Michele Angelo Di Bari, Claudia D’Agostino, Ilaria Vanni, Geraldina Riccardi, Stefano Marcon, Gabriele Vaccari, Barbara Chiappini, Sylvie L. Benestad, Umberto Agrimi, Romolo Nonno

**Affiliations:** 1 Department of Food Safety, Nutrition and Veterinary Public Health, Istituto Superiore di Sanità, Rome, Italy; 2 Norwegian Veterinary Institute, Ås, Norway; Creighton University, UNITED STATES

## Abstract

Prions are infectious agents that replicate through the autocatalytic misfolding of the cellular prion protein (PrP^C^) into infectious aggregates (PrP^Sc^) causing fatal neurodegenerative diseases in humans and animals. Prions exist as strains, which are encoded by conformational variants of PrP^Sc^. The transmissibility of prions depends on the PrP^C^ sequence of the recipient host and on the incoming prion strain, so that some animal prion strains are more contagious than others or are transmissible to new species, including humans. Nor98/atypical scrapie (AS) is a prion disease of sheep and goats reported in several countries worldwide. At variance with classical scrapie (CS), AS is considered poorly contagious and is supposed to be spontaneous in origin. The zoonotic potential of AS, its strain variability and the relationships with the more contagious CS strains remain largely unknown. We characterized AS isolates from sheep and goats by transmission in ovinised transgenic mice (tg338) and in two genetic lines of bank voles, carrying either methionine (BvM) or isoleucine (BvI) at PrP residue 109. All AS isolates induced the same pathological phenotype in tg338 mice, thus proving that they encoded the same strain, irrespective of their geographical origin or source species. In bank voles, we found that the M109I polymorphism dictates the susceptibility to AS. BvI were susceptible and faithfully reproduced the AS strain, while the transmission in BvM was highly inefficient and was characterized by a conformational change towards a CS-like prion strain. Sub-passaging experiments revealed that the main strain component of AS is accompanied by minor CS-like strain components, which can be positively selected during replication in both AS-resistant or AS-susceptible animals. These findings add new clues for a better comprehension of strain selection dynamics in prion infections and have wider implications for understanding the origin of contagious prion strains, such as CS.

## Author summary

Prions are transmissible agents responsible for fatal neurodegenerative diseases in humans and animals. Prions exist as strains, exhibiting distinct disease phenotypes and transmission properties. Some prion diseases occur sporadically with a supposedly spontaneous origin, while others are contagious and give rise to epidemics, mainly in animals. We investigated the strain properties of Nor98/atypical scrapie (AS), a sporadic prion disease of small ruminants. We found that AS was faithfully reproduced not only in a homologous context, i.e. ovinised transgenic mice, but also in an unrelated animal species, the bank vole. A natural polymorphism of the bank vole prion protein, coding for methionine (BvM) or for isoleucine (BvI) at codon 109, dictated the susceptibility of voles to AS, with BvI being highly susceptible to AS and BvM rather resistant. Most importantly, the M109I polymorphism mediated the emergence of AS-derived mutant prion strains resembling classical scrapie (CS), a contagious prion disease. Finally, by sub-passages in bank voles, we found that the main strain component of AS is accompanied by minor CS-like strain components, which can be positively selected during replication in both AS-resistant or AS-susceptible vole lines. These findings allow a better understanding of strain selection dynamics and suggest a link between sporadic and contagious prion diseases.

## Introduction

Transmissible spongiform encephalopathies (TSEs), or prion diseases, are fatal neurodegenerative disorders of humans and animals. Although TSEs can occur as sporadic, acquired or inherited (genetic) forms, they have been historically defined based on their experimental transmissibility.

In humans, most of TSEs have idiopathic (sporadic Creutzfeldt Jakob disease, variably protease sensitive prionopathy) or genetic origin (fatal familial insomnia, Gerstmann-Straussler-Scheinker disease, genetic Creutzfeldt Jakob disease). However, those that have raised the most serious public concerns are the acquired forms, with zoonotic (variant Creutzfeldt Jakob disease) or iatrogenic origin (iatrogenic Creutzfeldt Jakob disease) [1].

Animal TSEs present largely as acquired diseases: classical scrapie (CS) in small ruminants, bovine spongiform encephalopathy (BSE) in cattle, chronic wasting disease (CWD) in cervids, and, likely, the recently discovered prion disease of dromedary camels [2, 3]. However, the existence of sporadic or idiopathic forms in animals is increasingly recognized: H- and L-BSE in cattle [4, 5], Nor98/atypical scrapie (AS) in small ruminants [6] and, possibly, the newly discovered forms of CWD in some European cervid species [7–11] do not show an obvious infectious origin and are often supposed to be spontaneous.

Prion diseases are characterized by the accumulation in the central nervous system of PrP^Sc^, the post-translational misfolded form of the host-encoded cellular prion protein (PrP^C^). In contrast to PrP^C^, PrP^Sc^ is partially resistant to protease digestion, insoluble in non-denaturant detergents and rich in β-sheet structure [12]. Typically, following proteinase K (PK) digestion, PrP^C^ is fully hydrolysed while PrP^Sc^ is only partially cleaved at the amino terminal portion, leaving a PK-resistant core (PrP^res^), frequently characterized, in western blot analysis, by three bands corresponding to the unglycosylated, mono-glycosylated and di-glycosylated isoforms of PrP^Sc^.

According to the protein-only hypothesis [13], the infectious agent of TSEs is mainly or solely composed by PrP^Sc^, while the phenomenon of prion strain variability, revealed by the existence of different phenotypes of the same disease, would depend on different PrP^Sc^ conformations [14, 15].

The transmission of prions between species is limited by the transmission barrier. Early studies argued that it resides in PrP primary structure differences between donor and recipient species. Many studies support the view that prion propagation proceeds most efficiently when the interacting PrP^Sc^ and PrP^C^ are of identical primary structure, as shown by several instances in which the barrier was overcome by replacing the PrP gene of the recipient with its counterpart from the donor [16]. However, even if the PrP sequences of donor and recipient are identical, transmission barriers can still occur, as in the case of variant CJD from humans inoculated into mice expressing human PrP [17], suggesting that the barrier doesn’t depend only on the PrP sequences homology but also on the inoculated strain.

It has been proposed that prion populations are composed of a variety of conformational variants, or quasispecies; when the environment changes, the most efficiently replicating variants becomes the predominant components of the population, which then constitute a distinct sub-strain [18–20]. In this context, the PrP sequence of the recipient species would dictate the range of possible PrP conformations, hence conditioning the susceptibility to different prion strains, where the transmission barrier would reflect the selection of conformational variants [18].

Although the gold standard for the characterization of prion strains is still based on bioassay in rodents, several studies demonstrated that prion strains can be partially distinguished based on different biochemical properties of PrP^Sc^.

The electrophoretic features of PrP^res^ [14, 21, 22], the relative proteinase resistance of PrP^Sc^ [23, 24], or the physico-chemical behavior of PrP^Sc^ during denaturation [25–27] have been frequently used to discriminate prion strains.

Most prion diseases are characterized by PrP^res^ of around 27–30 kDa (PrP^res^ 27–30), but atypical PrP^res^ patterns have been described in both humans (Gerstmann-Sträussler-Scheinker disease and variably protease-sensitive prionopathy) and animals (AS) [28–35].

First recognized in Norwegian sheep in 1998 [34], AS has been identified in sheep and goats in most of Europe [6], Canada [36], USA [37], New Zealand and Australia [38, 39] and Japan [40]. AS differs from CS cases in the distribution of brain lesions, the pattern of PrP^Sc^ deposition, the electrophoretic signature in western blot and the association with polymorphisms of the PrP gene [34, 41, 42]. The different electrophoretic profiles of PrP^res^ from CS and AS cases implies that the PrP^Sc^ aggregates associated with these diseases have different conformations. Indeed, when treated with high concentration of PK, PrP^Sc^ from AS cases is cleaved at both N- and C-termini resulting in an atypical PK resistant core of ~8 kDa, in contrast to the classical, N-terminal cleaved PrP^res^ of 27–30 kDa from CS cases [35]. By using a milder PK treatment, it has been shown that PrP^res^ from AS cases might also present as a multiband pattern, where the small N- and C-terminally truncated PrP^res^ fragment is accompanied by at least two larger PrP^res^ fragments [34, 43].

Reported mainly in aged sheep and goats, AS occurs with a sporadic distribution, compatible with a non-contagious, possibly spontaneous, origin [6, 44–46].

Epidemiological evidences for natural transmission of AS are still lacking, although its transmissibility has been demonstrated experimentally in sheep (by intracranial and oral route) [47–50] and in transgenic mice expressing sheep PrP [51–55], porcine PrP [56] or bovine PrP [57]. Interestingly, low levels of infectivity were also detected in peripheral tissues of sheep with natural or experimental AS [49, 58].

In the present study, we investigated the transmissibility of AS in bank voles (*Myodes glareolus*), a wild type rodent model susceptible to a wide range of prion diseases [8, 55, 59–68]. By using two genetic lines of voles, either homozygous for methionine (BvM) or for isoleucine (BvI) at PrP codon 109, we found that the M109I polymorphism dictates the susceptibility of bank voles to AS and mediates the emergence of minor strain components with CS-like strain properties.

## Results

### Strain identity between Norwegian and Italian AS isolates

In order to investigate possible strain variation among AS Italian isolates, as well as to compare their strain properties with Norwegian Nor98/AS, we inoculated tg338 mice with Italian atypical scrapie isolates and with a previously characterized Norwegian AS isolate [51]. The details of the AS isolates used in this study are summarized in Table 1. Tg338 mice, which are known to be a reliable model for AS characterization [51, 53, 58, 69], allowed to compare the transmission characteristics of Italian and Norwegian isolates.

**Table 1:** Characteristics of natural atypical scrapie isolates used in the transmission studies.

Isolate (ID)*	Origin	Species	Genotype^¶^
Sh-N1	Norway (Lindås)	Sheep	ALRQ/ALHQ
Sh-N2	Norway (Hardbakke)	Sheep	AFRQ/ALHQ
Sh-N3	Norway (Rauland)	Sheep	AFRQ/AFRQ
Sh-I1	Italy	Sheep	ALRR/ALRR
Sh-I2	Italy	Sheep	AFRQ/AFRQ
Go-I1	Italy	Goat	ALRQ/ALHQ (240 S/P)
Go-I2	Italy	Goat	ALRQ/ALHQ

* The isolates were named based on species, geographical origin and serial number: sheep (Sh) or goat (Go), Norway (N) or Italy (I).

^**¶**^PrP genotype at sheep polymorphic codons 136, 141, 154 and 171. Although the positions 136 and 171 are not polymorphic in goat, the same nomenclature was also used for this species, with the addition of any difference with respect to the ovine wildtype sequence (Accession number AJ000739).

All the Italian AS isolates, deriving from sheep and goats with different PrP genotypes, transmitted the disease to tg338 mice with complete attack rates (Table 2).

**Table 2:** Transmission of atypical scrapie from sheep and goat isolates to tg338 mice.

Inoculum*	Passage	Incubation times (Dpi)^¶^	Attack rate^§^
Sh-I1	P1	224 ± 42	7/7
Sh-I2	P1	219 ± 19	7/7
	P2	195 ± 1	7/7
	P3	187 ± 17	12/12
Go-I1	P1	211 ± 14	7/7
	P2	190 ± 21	8/8
Go-I2	P1	239 ± 23	9/9
	P2	192 ± 9	11/11
Sh-N3	P1	196 ± 17	12/12
Sh-N3^BvI^	P1	198 ± 16	10/10

* The inoculum ID refers to the isolate from Table 1. The superscript (BvI) indicates that the inoculum derived from Sh-N3 after adaptation in BvI (two passages)

¶ The incubation times are expressed as mean of days post inoculation (dpi) ± standard deviation

^§^ Number of mice with confirmed TSE / number of mice inoculated. Confirmation of prion infection was assessed by western blotting for PrP^res^ and/or histopathology.

**Fig 1:**
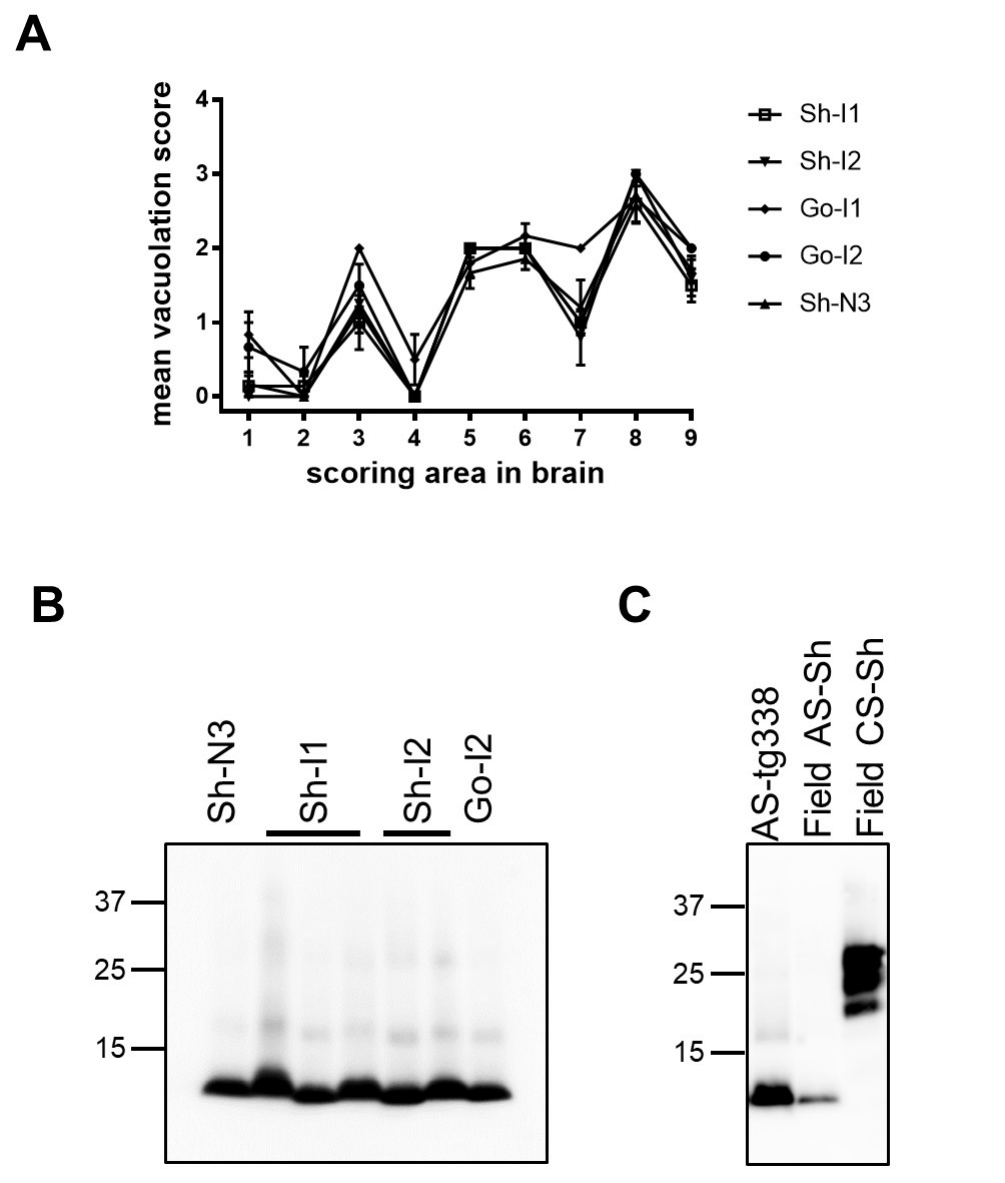
Distribution of spongiform neurodegeneration and PrP^Sc^ type in tg338 mice infected with AS isolates from Italian sheep and goats. (A) Lesion profiles in groups of tg338 mice infected with AS isolates reported in Table 2. Data points represent the mean ± SEM of at least five mice per group. Brain-scoring areas: medulla [1], cerebellum [2], superior colliculus [3], hypothalamus [4], thalamus [5], hippocampus [6], septum [7], retrosplenial and adjacent motor cortex [8], and cingulate and adjacent motor cortex [9]. (B) Representative western blot showing PK-treated PrP^Sc^ from brains of tg338 mice inoculated with the AS isolates (indicated on the top of the blot). The AS inocula are indicated as in Table 2. The positions of MW markers are indicated on the left of the blot (in kilodaltons). Membrane was probed with 9A2 monoclonal antibody. (C) Representative western blot showing PK-treated PrP^Sc^ from brains of tg338 mice inoculated with AS in comparison with sheep naturally infected with AS or CS (indicated on the top of the blot). The positions of MW markers are indicated on the left of the blot (in kilodaltons). Membrane was probed with 9A2 monoclonal antibody.

The transmission features of Italian isolates were uniform and indistinguishable from those of the Norwegian isolate regarding survival time, attack rate, lesion profiles, and PrP^res^ biochemical characteristics (Table 2 and Fig 1). At first passage, the mean survival times of tg338 ranged from 211 to 239 days post inoculation (dpi), slightly longer than what observed with the Norwegian isolate (196 dpi). This slight difference was probably due to different infectivity levels of the inocula. Indeed, at second passage, the mean survival time of Italian AS isolates ranged between 190 and 195 dpi and was retained at third passage (Table 2).

In agreement with previous findings in tg338 with AS [51, 53, 70], all AS isolates produced intense vacuolar and micro-vacuolar degeneration, mainly located in the superior colliculus, thalamus, hippocampus, septum and cerebral cortices, as shown by lesion profile (Fig 1A). Micro-vacuolation was also observed in the white matter, substantia nigra and caudate putamen.

Western blot analysis of PrP^res^ from the brain of tg338 mice showed the same electrophoretic profile for all cases, characterized by the expected low molecular weight PrP^res^ fragment of ~ 8 kDa (Fig 1B), indistinguishable from the PrP^res^ pattern observed in the sheep isolates analyzed with the same WB method (Fig 1C).

These results concur with the biochemical characterization of the AS isolates used in this study [35], showing that Italian and Norwegian AS isolates share the same strain features regardless of the host species, PrP genotype and geographical origin. This conclusion agrees with previous evidences showing no strain variation of European AS isolates [48, 51–53, 69].

### High susceptibility of BvI to atypical scrapie with preservation of strain characteristics

We previously showed that AS in small ruminants shares the atypical electrophoretic pattern of PrP^res^ with some rare human prion diseases, VPSPr and GSS [35]. Since bank voles carrying isoleucine at PrP codon 109 (BvI) are susceptible to human VPSPr and GSS [66, 67], we tested their susceptibility to AS isolates.

All AS isolates transmitted to BvI with similar survival times, ranging from 238 to 258 dpi, irrespective of the donor species, PrP genotypes and geographical origin (Table 3).

**Table 3:** Transmission of atypical scrapie isolates to BvI and BvM.

Inoculum*	BvI			BvM		
	Passage	Incubation times (Dpi)^¶^	Attack rate^§^	Passage	Incubation times (Dpi)^¶^	Attack rate^§^
Sh-I1	P1	247 ± 24	14/14	P1	>1064	0/11
	P2	241 ± 25	12/12			
Sh-I2	P1	238 ± 5	9/9	nd		
	P2	258 ± 25	12/12			
Sh-I2^tg338^	P1	266 ± 26	12/12			
Go-I2	P1	238 ± 34	12/12	nd		
Sh-N1	nd			P1	>1096	0/16
Sh-N2	nd			P1	661, 883	2/13
				P2	115 ± 6	11/11
				P3	114 ± 7	11/11
Sh-N3	P1	258 ± 50	11/11	P1	>936	0/9
	P2	257 ± 55	5/5			
	P3	237 ± 33	6/6			
Sh-N3^BvI^				P1	149, 224, 230, 399	4/8
				P2	89 ± 5	13/13
Sh-N2^BvM^	P1	96 ± 3	11/11			
	P2	86 ± 6	14/14			

nd, not done

* The inoculum ID refers to the isolate from Table 1. The superscripts indicate the animal model in which the isolate was adapted.

^¶^ The incubation times are expressed as mean days post inoculation (dpi) ± standard deviation. When no PrP^Sc^-positive animal was found in the group, survival time is shown as longer (>) than the survival time of the last culled/dead animal

^§^ Number of bank voles with confirmed TSE / number of bank voles inoculated. Confirmation of prion infection was assessed by western blotting and/or histopathology.

**Fig 2:**
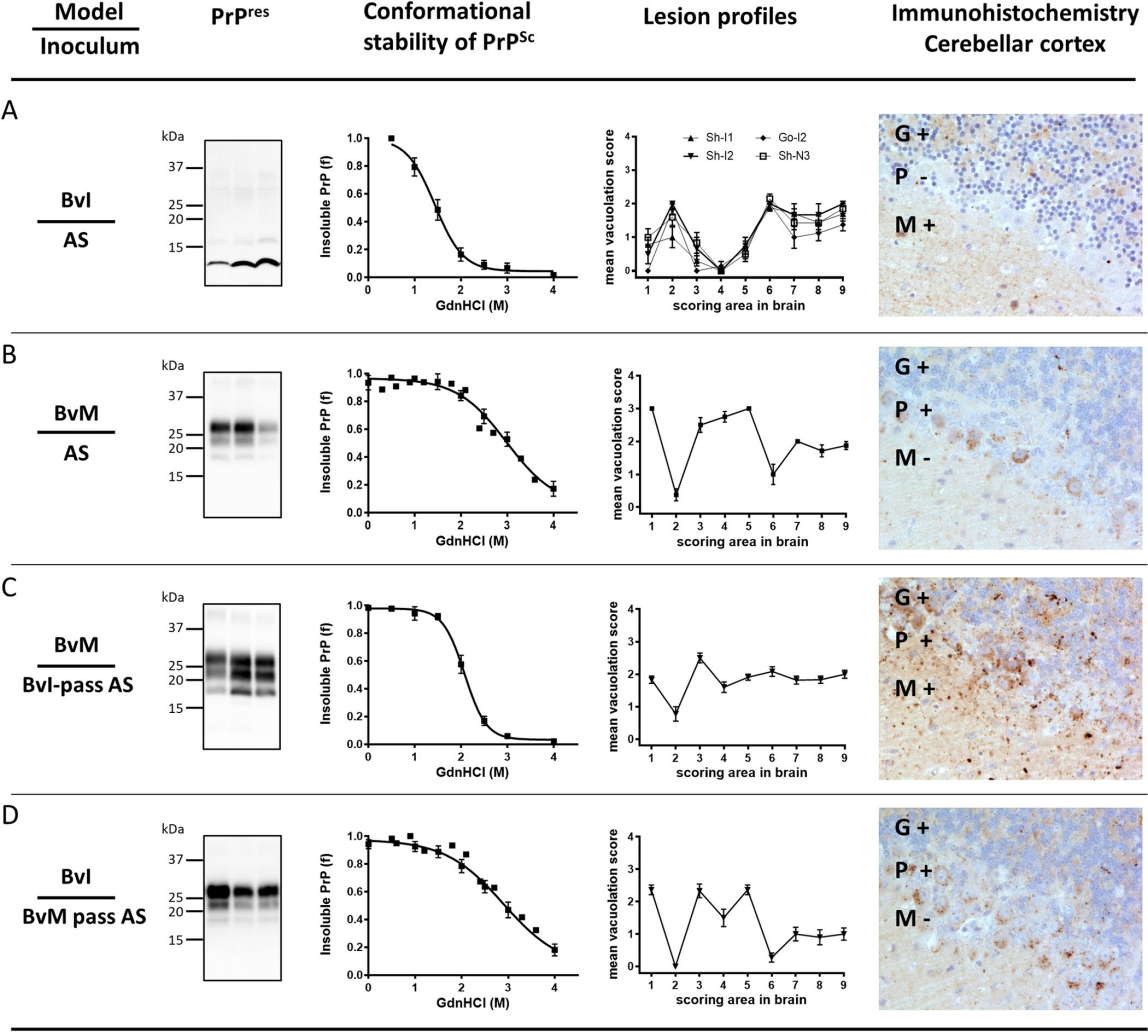
Transmission phenotypes in BvI and BvM infected with AS isolates. Biochemical properties of PrP^Sc^ (PrP^res^ typing and conformational stability) and neuropathological patterns (lesion profiles and immunohistochemistry for PrP^Sc^) in BvI inoculated with AS (A) or BvM-passaged AS (D) and in BvM inoculated with AS (B) or BvI-passaged AS (C), as indicated in the left column (model/inoculum). Western blots in the second column (PrP^res^ typing) show PK-treated PrP^Sc^ from representative brains of voles. In A, C and D the first lane was loaded with samples from first passages; all other samples were from second passage experiments. The positions of MW markers are indicated on the left of the blots (in kilodaltons). Membranes were probed with mAb 9A2. The conformational stability of PrP^Sc^ (third column) was analyzed by denaturation with increasing concentration of GdnHCl, followed by a solubility assay. The fraction of insoluble PrP^Sc^ was plotted as a function of GdnHCl concentration (M) and best-fitted using a four parameter logistic equation. Error bars represent the SEM of at least three animals per group: in A the curve merges two groups from different first passages (Sh-I1 and Sh-N3), while graphs in B, C and D show data derived from second passage experiments. Lesion profiles showing spongiform degeneration in groups of AS infected voles after primary transmission (A and D) or second passage (B and C). Data points represent the mean ± SEM of at least five voles per group. Brain scoring areas: medulla [1], cerebellum [2], superior colliculus [3], hypothalamus [4], thalamus [5], hippocampus [6], septum [7], retrosplenial and adjacent motor cortex [8], and cingulate and adjacent motor cortex [9]. Immunohistochemical detection of PrP^Sc^ in the cerebellar cortex was performed with SAF84 mAb. The presence of PrP^Sc^ in the granular layer (G), Purkinje cells (P) and molecular layer (M) of cerebellum is indicated (+, positive; -, negative).

Interestingly, the propagation of AS isolates in BvI was highly efficient, with 100% attack rate and apparent lack of transmission barrier, as showed by the absence of shortening of the survival times at the second and third passages (Table 3). Furthermore, BvI PrP^Sc^ was characterized by a small PrP^res^ internal fragment of ~8 kDa (Fig 2A), cleaved at both N- and C-termini (S1 Fig), and by a low conformational stability, with [GdnHCl]_1/2_ mean value of 1.5 M (Fig 2A). Thus, BvI PrP^Sc^ fully matched the biochemical properties of small ruminant AS PrP^Sc^, characterized by a low molecular weight internal PrP^res^ fragment and a low conformational stability (mean [GdnHCl]_1/2_ range 1.3–1.5 M) as previously described [27, 34, 35].

All AS isolates produced a similar distribution of vacuolar degeneration in the brain of BvI characterized by intense micro-vacuolar degeneration of the cerebellum, hippocampus, lateral septal nuclei, cerebral cortices (lesion profiles in Fig 2A). Micro-vacuolation was also observed in the white matter and substantia nigra.

By immunohistochemistry (IHC), intense PrP^Sc^ deposition was observed mainly in white matter and some gray matter areas (Figs 2A and S2). White matter deposition was characterized by a fine punctate pattern in the alveus (S2 Fig), stratum lacunosum-moleculare of CA1 (S2 Fig), dorsal tier of substantia nigra, internal capsule and putamen. Small and medium-sized irregular PrP^Sc^ deposits were also observed in the white matter, mainly in the reticular formation and vestibular nucleus. In the gray matter, fine granular PrP^Sc^ immunolabeling and aggregates were prominently observed in the geniculate nucleus, superior colliculus, hippocampus (S2 Fig) and medial layers of cortices. Finally, small PrP^Sc^ deposits of irregular shape were detected in the cerebellar granular layer, while punctate and linear patterns characterized the molecular layer (Fig 2A). Intracellular PrP^Sc^ deposition was not observed. Remarkably, the pathological phenotype observed in BvI matches that described in small ruminant AS, with the main involvement of rostral areas, distinctive immunolabeling in white matter and lack of intracellular PrP^Sc^ deposition [34, 70, 71].

To verify the preservation of AS strain properties after passage in BvI, we transmitted BvI-adapted AS to tg338 mice (Table 2). Interestingly, this transmission was indistinguishable from those of AS field isolates in the same tg338 model, as for incubation time, lesion profile and PrP^res^ characteristics (Table 2, S3 Fig).

Finally, tg338-adapted AS transmitted as efficiently as field AS cases to BvI and reproduced the same strain features, showing that bank voles with isoleucine at PrP codon 109 are also susceptible to AS deriving from a VRQ sequence (as expressed in tg338) (see Sh-I2^tg338^ in Table 3 and S3C Fig).

Overall, these data show that AS isolates transmit very efficiently to BvI and faithful preserve their strain characteristics.

### Inefficient propagation and strain shift of atypical scrapie in BvM

We previously reported the lack of transmission of AS isolates from Norway and UK in bank voles carrying methionine at PrP codon 109 (BvM) [55, 72]. In this study, we challenged BvM with an AS case from Italy (Sh-I1) and with an additional case from Norway (Sh-N3). We also report the final results from two Norwegian AS isolates (Sh-N1 and Sh-N2), whose interim negative results at 600 dpi have already been reported [72] (Table 3).

The transmission of AS isolates to BvM was inefficient, with most of the inocula resulting in the absence of clinical signs, TSE-associated vacuolation or PrP^res^ accumulation within the life span of the voles (Table 3). Only one isolate (Sh-N2) showed partial transmission in BvM, with two TSE-affected animals at 661 and 883 dpi (Table 3). These two positive BvM accumulated a PrP^Sc^ type characterized by PrP^res^ 27–30 instead of the expected 8 kDa PrP^res^ (S4 Fig). The second passage showed a dramatic shortening of the survival time, suggesting that a high transmission barrier limited the propagation of AS in BvM. The PrP^Sc^ type was preserved on second passage and was characterized by a 27–30 kDa PrP^res^ with the unglycosylated band of ~18 kDa (Fig 2B) and by high conformational stability, with [GdnHCl]_1/2_ mean value of 3.0 M (Fig 2B). Pathological assessment of standard areas in the brain showed large and medium vacuoles mainly localized in the thalamus, medulla, superior colliculus, hypothalamus and in all nuclei of mesencephalon, with no involvement of the white matter. The hippocampus showed rare vacuoles, and the cerebellum was not affected by spongiform change (Fig 2B). PrP^Sc^ deposition was mainly detected in the grey matter areas of thalamus, geniculate nucleus, superior colliculus and mesencephalon. In all these areas, intraglial and intraneuronal PrP^Sc^ depositions were the main patterns observed, frequently accompanied by diffuse and punctate immunolabeling in the neuropil (see the red nucleus in S2 Fig). In the cerebellum, fine granules of PrP^Sc^ were observed within Purkinje cells and rare granular deposits were found in the granular layer (Fig 2B).

Overall, we conclude that BvM are rather resistant to AS but, when infected, they propagate a strain with CS-like features, i.e. accumulation of PrP^res^ 27–30 mainly in grey matter areas of the brainstem, hindbrain and diencephalon.

### The inability of BvM to reproduce the atypical scrapie strain

We then attempted to overtake the low susceptibility of BvM to AS by inoculating a group of BvM with the AS strain previously adapted in BvI, reported as Sh-N3^BvI^ in Table 3. As previously observed with natural isolates, BvM were barely susceptible to BvI-adapted AS showing a partial attack rate (Table 3).

At first passage, the mean survival times ranged from 149 to 399 dpi with 50% attack rate (Table 3). The survival time decreased dramatically at the second passage (mean of 89 dpi), indicating a significant transmission barrier even when AS has been previously propagated in BvI, i.e. differing only at codon 109 of the PrP sequence.

Overall, the neuropathological and molecular phenotypes that typify AS were not preserved in BvM after two passages with BvI-adapted AS. The electrophoretic pattern of PrP^res^ was characterized by PrP^res^ 27–30 and the conformational stability analysis of PrP^Sc^ showed a resistance to denaturation intermediate between BvI- and BvM-adapted AS, with [GdnHCl]_1/2_ mean value of 2.0 M (Fig 2C). Neurodegeneration was characterised by medium and small vacuoles in medulla, superior colliculus, hypothalamus, thalamus, hippocampus, septum, thalamic nuclei, retrosplenial and cingulate cortices (Fig 2C). Spongiform degeneration was not observed in white matter areas and substantia nigra. PrP^Sc^ deposits were observed in grey matter areas of the hippocampus (S2 Fig), thalamus, mesencephalon (S2 Fig), medulla, cerebellum (Fig 2C) and cortices. In the cerebellum, strong PrP^Sc^ immunolabeling involved the molecular, granular and Purkinje cells layers (Fig 2C). PrP^Sc^ was detected as intraglial and intraneuronal patterns, accompanied by punctate, granular and plaque-like deposits (Figs 2C and S2).

In contrast to the inefficient and unfaithful transmission of BvI-adapted AS to BvM, BvI were highly susceptible to BvM-adapted AS (survival time of 96 dpi, Table 3), showing pathological features, lesion profile, PrP^res^ type and PrP^Sc^ conformational stability overlapping those observed in BvM and distinct from BvI-adapted AS (Figs 2D and S2).

We conclude that BvM are unable to reproduce the AS strain characteristics either when AS originate from the natural host or after adaptation of AS in BvI. In both cases, BvM propagated CS-like variants, which were different from each other and from BvI-adapted AS and did not revert to AS-like features upon back passage in BvI. Indeed, BvI were susceptible to and faithfully reproduced the BvM-adapted AS strain, showing that the AS strain stably mutated upon replication in BvM.

### Emergence of PrP^res^ 27–30 in BvI

The finding that the AS strain mutates when propagated in BvM could depend on mutational events driven by AS propagation in BvM or might reflect pre-existing minor strain variants in AS that are positively selected in an animal model unable to reproduce the main AS strain. Interestingly, the AS-derived CS-like strain was efficiently and faithfully transmitted from BvM to BvI (Fig 2D), suggesting that BvI could have been able to reproduce a minor component if it was present in the original inoculum. We thus closely re-analyzed all AS-infected BvI looking for possible variant PrP^res^ patterns.

Out of 93 BvI investigated, all but one showed the expected 8 kDa PrP^res^. A single BvI showed a mixed PrP^res^ pattern, characterized by the presence of PrP^res^ 27–30 accompanied by small amounts of 8 kDa PrP^res^ (S5 Fig). This single BvI showed the shortest survival time in its group (second passage of Sh-N3). In its brain, we observed microvacuoles in the white matter and hippocampus, as observed in AS-affected BvI, and large and medium vacuoles in the thalamus as seen in BvM. The presence of a mixed phenotype was then confirmed by IHC (S5 Fig), which showed both intracellular PrP^Sc^ deposition in grey matter, a feature observed in BvM-adapted AS but not in BvI-adapted AS, as well as an AS-like PrP^Sc^ deposition in the hippocampus and cerebellum. Brain homogenate from this vole was inoculated in both BvM and BvI lines.

**Fig 3:**
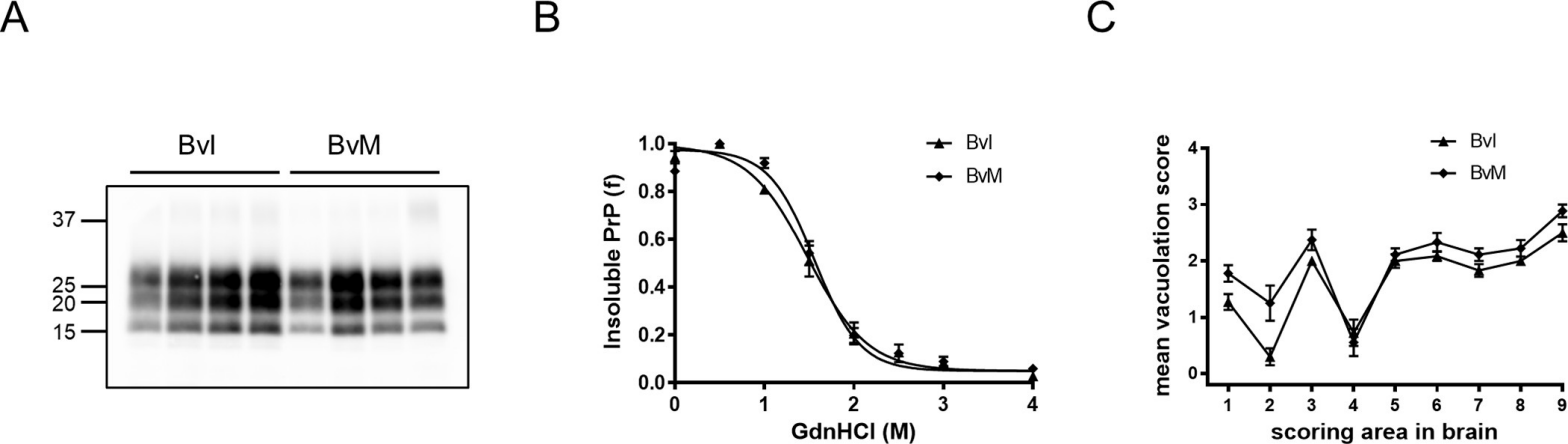
Neuropathological and PrP^Sc^ phenotypes induced by the mixed case after passage in BvI and BvM. (A) Representative western blot showing PK-treated PrP^Sc^ from brains of Bv inoculated with the mixed case emerged in BvI. The recipient model is indicated on the top of the blot. The positions of MW markers are indicated on the left of the blot (in kilodaltons). Membrane was probed with 9A2 monoclonal antibody. (B) The conformational stability of PrP^Sc^ was analyzed by denaturation with increasing concentration of GdnHCl, followed by a solubility assay. The fraction of insoluble PrP^Sc^ was plotted as a function of GdnHCl concentration (M) and best-fitted and using a four parameter logistic equation. Error bars represent the SEM of data from analyses of at least three animals per group. (C) Comparison of lesion profiles in BvI and BvM infected with the mixed case. Data points represent the mean ± SEM of at least five voles per group. Brain-scoring areas: medulla [1], cerebellum [2], superior colliculus [3], hypothalamus [4], thalamus [5], hippocampus [6], septum [7], retrosplenial and adjacent motor cortex [8], and cingulate and adjacent motor cortex [9].

Unlike previous transmission of BvI-adapted AS (Table 3), the transmission of this mixed case to BvM was highly efficient, with short survival times (74 ± 6 dpi) and 100% attack rate (15/15 voles). PrP^Sc^ was characterized by low conformational stability (GdnHCl_1/2_ mean value of 1.6 M) and PrP^res^ 27–30 (Fig 3). The regional distribution of spongiform degeneration partially overlapped that observed in BvM inoculated with BvI-adapted AS (compare lesion profiles in Figs 3C and 2C) with preservation of white matter areas and substantia nigra. At variance with BvI-passaged AS, the second passage of the mixed case in BvI showed a transmission barrier, with shortening of the survival time from 170 dpi of the donor BvI to 109 ± 5 dpi, a mean survival time much shorter than expected for BvI-adapted AS (237–258 dpi, Table 3). The molecular and neuropathological phenotypes in BvI overlapped those observed in BvM (Fig 3). All recipient BvI accumulated PrP^res^ 27–30, although minute amounts of 8 kDa PrP^res^ were still observed in 2 out of 12 BvI.

From these experiments, we conclude that, although very rarely, an AS-derived mutant strain characterized by CS-like features can emerge in BvI, a model able to propagate AS efficiently. The mutant strain co-existed with bona fide AS and was positively selected upon subpassaging in BvM and BvI, probably due to its disease kinetics faster than bona fide AS.

## Discussion

Although it is unclear if AS has a spontaneous or acquired origin, AS isolates have been successfully transmitted in transgenic mice expressing sheep/goat-derived PrP sequences [51–54, 69, 73], in sheep [47–50] and, with a much lower efficiency, in transgenic mice expressing bovine [57] or porcine PrP [56]. Wild type inbred mouse lines were repeatedly shown to be resistant to experimental challenge with AS isolates [34, 51, 53, 72]. The picture emerging from these studies shows that AS faithfully propagated on sheep/goat PrP sequences, while in non-small ruminant PrP-bearing hosts a high transmission barrier was accompanied by inability to efficiently reproduce AS prions and by strain mutation, with a shift from PrP^res^ of 8 kDa to PrP^res^ 27–30 [56, 57]. In one instance, AS strain mutation has been observed even upon intra-species intracerebral challenge, where a single sheep showed a strain shift to the classical scrapie strain CH1641 [74].

In this study, we tested the transmissibility of AS in two genetic lines of bank voles, a rodent species known to be susceptible to a wide range of prion strains [8, 55, 59–68].

Unexpectedly, our findings were reminiscent of previous transmission studies with AS, as we observed the same dichotomy between a susceptible recipient model, BvI, able to faithfully reproduce AS prions, and the more resistant BvM, in which rare positive transmissions were characterized by strain mutation and the accumulation of PrP^res^ 27–30 instead of 8 kDa PrP^res^. Thus, these data add to previous evidence showing that, in animal models unable to reproduce AS prions, long lasting and inefficient primary transmissions of AS isolates mediate the emergence of mutant prion strains, raising the question of their origin. Do they derive from the conversion of PrP^Sc^ to alternative conformations caused by PrP amino acid mismatches between donor and recipient host species, as posited by the “deformed templating” model [75], or are minor strain variants pre-existing in AS isolates and positively selected in animal species unable to reproduce bona fide AS prions, as postulated by the “conformational selection” model [18]? The recent experiments by Huor and colleagues [57] represent a cornerstone in support of the latter hypothesis, as they used an ultrasensitive *in vitro* replication method to directly demonstrate the presence of classical BSE prions as a minor component in AS isolates, which can be positively selected in transgenic mice expressing bovine PrP. Accordingly, Huor and colleagues also found that AS is accompanied by minute amounts of classical BSE prions even during replication in tg338 mice, a sheep-based transgenic mouse line. We consider that our results in bank voles can be better interpreted within the framework of the conformational selection model, thus supporting the hypothesis that minor strain variants are present in AS isolates.

The transmission of AS in BvI was highly efficient and without obvious transmission barrier, as secondary transmission did not lead to shortening of the survival time. Most importantly, BvI faithfully recapitulated the pathological features reported in small ruminants AS, including N- and C- terminal cleaved 8 kDa PrP^res^ [34, 35, 72, 76, 77], PrP^Sc^ with very low conformational stability [27, 35], brain distribution of spongiform degeneration and PrP^Sc^ deposition mainly in neocortex, hippocampus, basal ganglia and cerebellum [6], involvement of white matter tracts and absence of intraneuronal PrP^Sc^ deposits [6, 71]. Accordingly, bioassay of BvI-adapted AS in tg338 reporter mice confirmed the full preservation of the AS strain in BvI. Thus, despite the rather diverging PrP sequence compared to sheep, BvI PrP was fully able to acquire the PrP^Sc^ conformation that characterize AS, behaving as expected for sheep/goat-derived PrPs and allowing disease kinetics similar or even faster than in transgenic mice over-expressing small ruminant PrPs [51–54, 69, 73]. These results prove that the small ruminant PrP sequence is not an absolute requirement for propagation of bona fide AS and that amino acid mismatches with sheep PrPs did not cause deformed templating of AS in BvI.

Unexpectedly, the simple met/ile substitution at PrP codon 109 in BvM made voles unable to reproduce bona fide AS, so that BvM behaved very similarly to other non-small ruminant PrP-bearing animal models. Despite the resistance of BvM, a single AS isolate induced disease in two voles at first passage, with a clear phenotype shift. The shifted phenotype was maintained at the second and third passages, leading to a BvM-adapted strain with CS-like features, such as PrP^res^ 27–30, main subcortical involvement and intraneuronal PrP^Sc^ deposition in grey matter. In keeping with what observed in sheep [74], the AS-derived strain that emerged in BvM showed incubation time and neuropathological features reminiscent of BvM-adapted CH1641 [78]. In contrast, it is not surprising that classical BSE did not emerge in BvM, given the very low amounts of classical BSE prions detected in AS isolates [57] and the low susceptibility of BvM to classical BSE [59, 68]. In summary, the results in BvM are difficult to explain with deformed templating mechanisms. Indeed, the same or similar, CH1641 CS strain emerged in sheep and BvM, regardless of their diverging PrP sequences. Furthermore, as AS breed true in BvI, deformed templating in BvM could have only depended on the M109 amino acid residue, which is nonetheless the same of the corresponding position in sheep PrP (M112).

To find direct evidence that strain components characterized by PrP^res^ 27–30 could be propagated in a context favoring AS replication, we then looked for the presence of PrP^res^ 27–30 in all AS-affected BvI and found a single vole brain showing both 27–30 and 8 kDa PrP^res^. This finding is reminiscent of rare AS cases reported in sheep showing co-existence of CS and AS, either evinced by bioassay [79], or detected by WB [80], similarly to our finding in BvI. Furthermore, it demonstrates that PrP^res^ 27–30 components can propagate in BvI along with bona fide AS and can become the main component due to positive selection during experimental transmissions. Sub-passaging of the mixed case led to a BvI-adapted strain characterized by survival times shorter than bona fide AS and by PrP^res^ 27–30, thus supporting a positive selection mechanism. The presence of minimal amounts of PrP^res^ 8 kDa only in two BvI from this experiment can be explained by the three times shorter survival time of the mutant strain compared to bona fide AS, which didn’t allow PrP^res^ 8 kDa to replicate at detectable levels in all recipient BvI.

How a single amino acid variation in bank vole PrP sequence could determine the susceptibility to AS prions was further investigated through the inoculation of BvI-adapted AS into BvM. The phenotype obtained was different from the AS strain observed in BvI, showing that BvM was not able to reproduce the original AS strain properties regardless of whether the inoculum derived from natural hosts or BvI. The transmissions of BvI-adapted bona fide AS (i.e. without detectable phenotype shift) and of the mixed case to BvM led to the emergence of similar, although not identical, mutant PrP^res^ 27–30 strains. Interestingly, the transmission of BvI-adapted AS was much less efficient than that originating from the mixed case, with incomplete attack rate and much longer survival times (284 *vs* 74 dpi), which is in agreement with the presence of PrP^res^ 27–30 at detectable levels in the mixed case, but not in bona fide AS. Overall, these findings suggest that the inefficient transmission of BvI-adapted bona fide AS to BvM depends on the inability of BvM to replicate AS, while the accompanying strain mutation reflects the presence of minor prion variants, along with bona fide AS, in AS-affected BvI, as also observed in sheep [74] and tg338 mice [57].

Bank voles are the first wild type rodents showing susceptibility to AS. The peculiar susceptibility of bank voles to a wide range of prion sources has been mainly ascribed to features of vole PrP rather than to the vole genetic background [59, 63, 68, 81–83]. Indeed, the ability of bank voles to faithfully propagate AS was clearly dependent on a single PrP amino acid residue, i.e. I109. This finding represents a clear parallel with previous experiments showing that I109 in Bv PrP was key for the faithful propagation of human prion diseases characterized by PrP^Sc^ with atypical features similar to AS [35], such as GSS and VPSPr [66, 67, 84]. This suggests a specific role of this amino acid residue, located in the central region of PrP, in the ability to replicate atypical PrP^Sc^ conformations characterised by N- and C-terminal cleaved PrP^res^ types. In AS natural hosts, the key role of single amino acid residues in this PrP region is highlighted by the strong association of the disease with specific sheep and goat PrP polymorphisms, such as F141 and H154 [41, 42]. Interestingly, small ruminants encode for methionine at codon 112, the corresponding of codon 109 of bank vole PrP, but this does not interfere with the susceptibility of natural hosts to AS. This sharp divergence might suggest that I109 plays a role exclusively in the vole PrP context, or that small ruminant PrP polymorphisms that are strongly associated with the development of AS in sheep, i.e. F141 and H154, allow to overtake a hypothetical inhibitory role of M109. Recent experiments favor the latter hypothesis, as transgenic mice expressing sheep PrP with isoleucine instead of methionine at codon 112 (corresponding to bank vole codon 109) are more susceptible to AS than those expressing wild type sheep PrP and develop spontaneous AS within the mice lifespan (Enric Vidal, personal communication). Overall, these considerations support the view that the central region of PrP plays a key role for the development, the transmissibility and the strain features of prions [85–89].

In conclusion, we show that BvI are uniquely susceptible to AS and are the first available wild type rodent model for experimental studies with this prion strain. The resistance of BvM to AS and the emergence of CS-like strains in this bank vole genetic line can be explained in the context of the conformational selection model, supporting evidences that AS isolates are composed of an ensemble of prion strain components, i.e. PrP^Sc^ conformations, with a main component characterized by 8 kDa PrP^res^ and minor components by PrP^res^ 27–30. Although it is a minor component, PrP^res^ 27–30 can still play an important role as it might be endowed with the contagiousness that is typical of CS and can be positively selected under specific circumstances, as we have shown here in BvI. Recent data showing that classical BSE prions are present in AS isolates led to suggest that AS cases might be a possible source of classical BSE prions [57]. Thus, the present findings add new clues for a better comprehension of strain selection dynamics and transmission barrier and might have wider implications for public health, namely for understanding the origin of the more contagious prion strains, such as CS.

## Materials and methods

### Ethics statement

Experiments involving animals adhered to the guidelines contained in the Italian Legislative Decree 116/92, which transposed the European Directive 86/609/EEC on Laboratory Animal Protection, and then in the Legislative Decree 26/2014, which transposed the European Directive 2010/63/UE on Laboratory Animal Protection. The experimental protocols were approved and supervised by the Service for Biotechnology and Animal Welfare of the Istituto Superiore di Sanità, and were authorized by the Italian Ministry of Health (decree numbers 84/12.B and 1119/2015-PR).

### Animal models

Bank voles carrying methionine (BvM) or isoleucine (BvI) at the polymorphic PRNP codon 109 and tg338 transgenic mice that overexpress the VRQ allele of ovine PrP were obtained from the breeding colony of Istituto Superiore di Sanità (ISS).

### Nor98/atypical scrapie isolates

Nor98/atypical scrapie (AS) isolates from sheep and goat were identified through active or passive surveillance programs in Italy and Norway. Most of the AS isolates were previously characterized for their PrP^Sc^ biochemical properties [35].

### PrP genotype of sheep and goat isolates

DNA extraction, PCR amplification and sequencing were carried out as previously reported [90]. Briefly, PrP coding sequence was amplified using F1 (5’-CATTTATGACCTAGAATGTTTATAGCTGATGCCA-3’) and R1 (5’- TTGAATGAATATTATGTGGCCTCCTTCCAGAC-3’) primers, with standard PCR conditions. Sequencing reactions were carried out with primers T1 (5’-GGTCCTCATAGTCATTGCC-3’), T2 (5’-TGGTGGCTACATGCTGGG-3’), T3 (5’-TTTACGTGGGCATTTGATGC-3’) and T4 (5’-GGCTGCAGGTAGACACTCC-3’) using Big Dye Terminator Cycle sequencing Kit v1.1 (Life Technologies), subsequently purified using BigDye XTerminator Purification Kit and detected with ABI PRISM 3130 apparatus (Life Technologies). Sequences were analyzed using the software Seq Scape v2.5 (Life Technologies).

### Bioassay

For the preparation of the inocula, brain tissues from TSE affected animals were homogenized at 10% (w/v) in phosphate buffered saline (PBS) and stored at -80°C. Groups of six-to-eight week old rodents were inoculated intracerebrally with 20μl of homogenate into the left cerebral hemisphere, under ketamine anaesthesia (ketamine 0.1μg/g). All animals were individually identified by a passive integrated transponder. Animals were examined twice a week until neurological signs appeared, after which they were examined daily. Animals were culled with carbon dioxide before neurological impairment was such as to compromise their welfare, in particular their ability to drink and feed adequately. At post-mortem, brains from inoculated mice were removed and divided sagittally. Half brain was frozen for WB analysis of PrP^Sc^ and half fixed in formol-saline for histological analysis. The attack rate was calculated as the number of animals scoring positive at post-mortem/number inoculated. For primary transmissions, animals found dead or culled for intercurrent disease before 200 dpi and scoring negative at postmortem were excluded from analyses. The survival time for animals scoring positive at post-mortem was calculated as the time from inoculation to culling or death.

### PrP^res^ analysis and western blotting

Brain homogenates (20% w/v) were prepared as previously described [64].

After adding an equal volume of 100 mM Tris-HCl containing 4% sarkosyl, the homogenates were incubated for 30min at 37°C with gentle shaking. Proteinase K (Sigma-Aldrich) was added at a final concentration of 50 μg/ml and then the samples were incubated for 1 h at 55°C with gentle shaking. Protease treatment was stopped with 3mM PMSF (Sigma-Aldrich). Aliquots of samples were added with an equal volume of isopropanol/butanol (1:1 v/v) and centrifuged at 20,000 g for 5min. Supernatants were discarded and the pellets were resuspended in denaturing sample buffer (NuPAGE LDS Sample Buffer, Invitrogen) and heated for 10 min at 90°C.

After electrophoresis on 12% bis-Tris polyacrylamide gels (Invitrogen) and WB on polyvinylidene fluoride membranes using the Trans-Blot Turbo Transfer System (Bio-Rad), the blots were processed with anti-PrP mAbs by using the SNAP i.d. 2.0 system (Millipore). Membranes were probed with mAbs SAF84 (aa 167–173, sheep numbering), 9A2 (aa 102–104) or 12B2 (aa 93–97). The PrP was visualized by enhanced chemiluminescent substrate (SuperSignal Femto, Pierce) and the ChemiDoc imaging system (Bio-Rad). The chemiluminescent signal was quantified by Image Lab software (Bio-Rad).

### Conformational stability analysis

The conformational stability of PrP^Sc^ in brain homogenates from bank voles was analysed by CSSA as previously described [27]. Briefly, aliquots of brain homogenates (6% w/v in 100 mM TrisHCl, pH 7.4) were added with an equal volume of 100 mM TrisHCl (pH 7.4), sarcosyl 4% and incubated for 1 h at 37°C with gentle shaking. Then, aliquots of each sample were incubated for 1 h at 37°C with different concentrations of GdnHCl (Pierce) to obtain final concentrations ranging from 0 to 4 M. After GdnHCl treatment samples were centrifuged at 20,000 g for 1 h at 22°C and the pellets were re-suspended in denaturing sample buffer (NuPage LDS Sample Buffer and NuPage Sample Reducing Agent, Invitrogen) and analysed by western blot. The dose-response curves allowed us to estimate the concentration of GdnHCl able to solubilize 50% of PrP^Sc^ (GdnHCl_1/2_). Individual denaturation curves were analyzed and best-fitted by plotting the fraction of PrP^Sc^ remaining in the pellet as a function of GdnHCl concentration, and using a four parameter logistic equation (GraphPad Prism).

### Neuropathology and immunohistochemistry

Histopathology and immunohistochemistry were carried out on formalin-fixed tissues as previously described [60]. Briefly, coronal brain sections were obtained from four antero-posterior levels including the following: 1) telencephalon at midlevel of caudate nucleus, 2) diencephalon at midlevel of thalamus, 3) midbrain, and 4) hindbrain at midlevel of medulla and cerebellum. Histological sections from the above levels were prepared and stained either with hematoxylin and eosin to assess spongiosis or were subjected to immunohistochemistry using the monoclonal antibody SAF84 as described [60]. Neuropathological assessment was performed on sections stained with hematoxylin and eosin, and lesion profiles were constructed scoring the vacuolar degeneration in nine gray-matter areas of the brain [60]. Vacuolation scores were derived from at least five individual animals per group and are reported as means ± SEM. Immunohistochemistry, was performed with mAb SAF84 on coronal sections, using the protocol previously described.

## Supporting information

S1 Fig Epitope mapping of PrP^res^ from voles infected with classical and atypical scrapie. Representative blots of PrP^res^ from brains of BvI inoculated with classical (CS) or atypical scrapie (AS). Replica blots were probed with 12B2, 9A2 and SAF84 mAbs. The positions of MW markers are indicated on the left of the blot (in kilodaltons).
(TIF)

S2 Fig Immunohistochemical features of BvI and BvM infected with AS isolates. PrP^Sc^ deposition patterns observed in hippocampus and red nucleus of BvI and BvM inoculated with AS and with AS-passaged in BvM and BvI, as indicated. Note that AS in BvI showed a fine punctate pattern in the alveus and stratum lacunosum-moleculare (Slm) of CA1 and no labeling in the red nucleus, while AS in BvM was characterized by intraglial and intraneuronal PrP^Sc^ depositions in the red nucleus, accompanied by diffuse and punctate immunolabeling in the neuropil. PrP^Sc^ deposits in BvM inoculated with BvI-passaged AS were mainly intraglial and intraneuronal in the hippocampus and red nucleus, accompanied by punctate, granular and plaque-like deposits in the red nucleus. Finally, PrP^Sc^ deposits in BvI inoculated with BvM-passaged AS overlapped with those observed in BvM inoculated with AS, mainly characterized by by intraglial and intraneuronal PrP^Sc^ depositions.
(TIF)

S3 Fig Preservation of AS strain properties after passage in BvI. (A) Lesion profiles in groups of tg338 mice infected with AS natural isolates and BvI-passaged AS (Sh-N3 BvI). (B) Representative western blot showing PrP^res^ from brains of tg338 infected with AS from sheep (AS-Sh) or with BvI-adapted AS (AS-BvI). (C) Lesion profiles in groups of BvI infected with AS natural isolates or with tg338-adapted AS (Sh-I2 tg338).
(TIF)

S4 Fig Primary passage of AS in BvM. Representative western blot showing PK-resistant PrP^Sc^ from brains of BvM infected with AS isolates Sh-N1 and Sh-N2. As with the others AS isolates, most BvM were negative. The positive BvM shown is one of the two only BvM, both inoculated with Sh-N2, resulting in positive transmission. Note that PrP^Sc^ in BvM inoculated with AS is characterized by PrP^res^ 27–30, similar to that in BvM inoculated with CS (control lanes) and different from AS in small ruminants and BvI. Membrane was probed with SAF84 mAb. The positions of MW markers are indicated on the left of the blot (in kilodaltons).
(TIF)

S5 Fig Biochemical and immunohistochemical features of the mixed case emerged at second passage of Sh-N3 in BvI. (A) Western blot of PrP^res^ from the brain of individual BvI inoculated with Sh-N3 (second passage), Sh-N3 (third passage) or heathy controls, as indicated. The BvI from the second passage of Sh-N3 shows the classical 27–30 PrP^res^ accompanied by small amount of the 8 kDa PrP^res^ (indicated with the arrow in long exposure blot on the right). Membrane was probed with 12B2 mAb. (B) Immunohistochemical analysis of the mixed case highlights the simultaneous presence of intracellular PrP^Sc^ deposits in CA1, characteristic of BvM inoculated with AS, and fine punctate deposits in the alveus, characteristic of BvI inoculated with AS.
(TIF)
