# Supplementary figures and images for: A single amino acid residue in bank vole prion protein drives permissiveness to Nor98/atypical scrapie and the emergence of multiple strain variants
Source: PLoS Pathog. 2022 Jun 22;18(6):e1010646. doi: 10.1371/journal.ppat.1010646 (PMC9255773; doi:10.1371/journal.ppat.1010646)

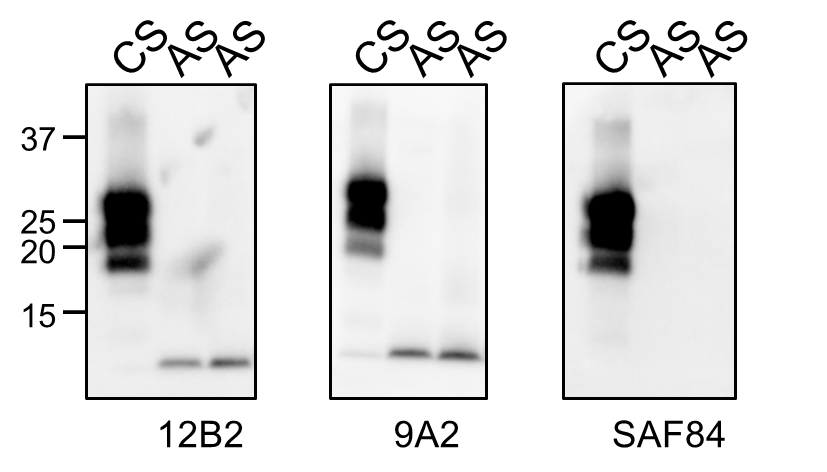

Supplement: S1 Fig — Representative blots of PrPres from brains of BvI inoculated with classical (CS) or atypical scrapie (AS). Replica blots were probed with 12B2, 9A2 and SAF84 mAbs. The positions of MW markers are indicated on the left of the blot (in kilodaltons). (TIF) [file ppat.1010646.s001.tif]

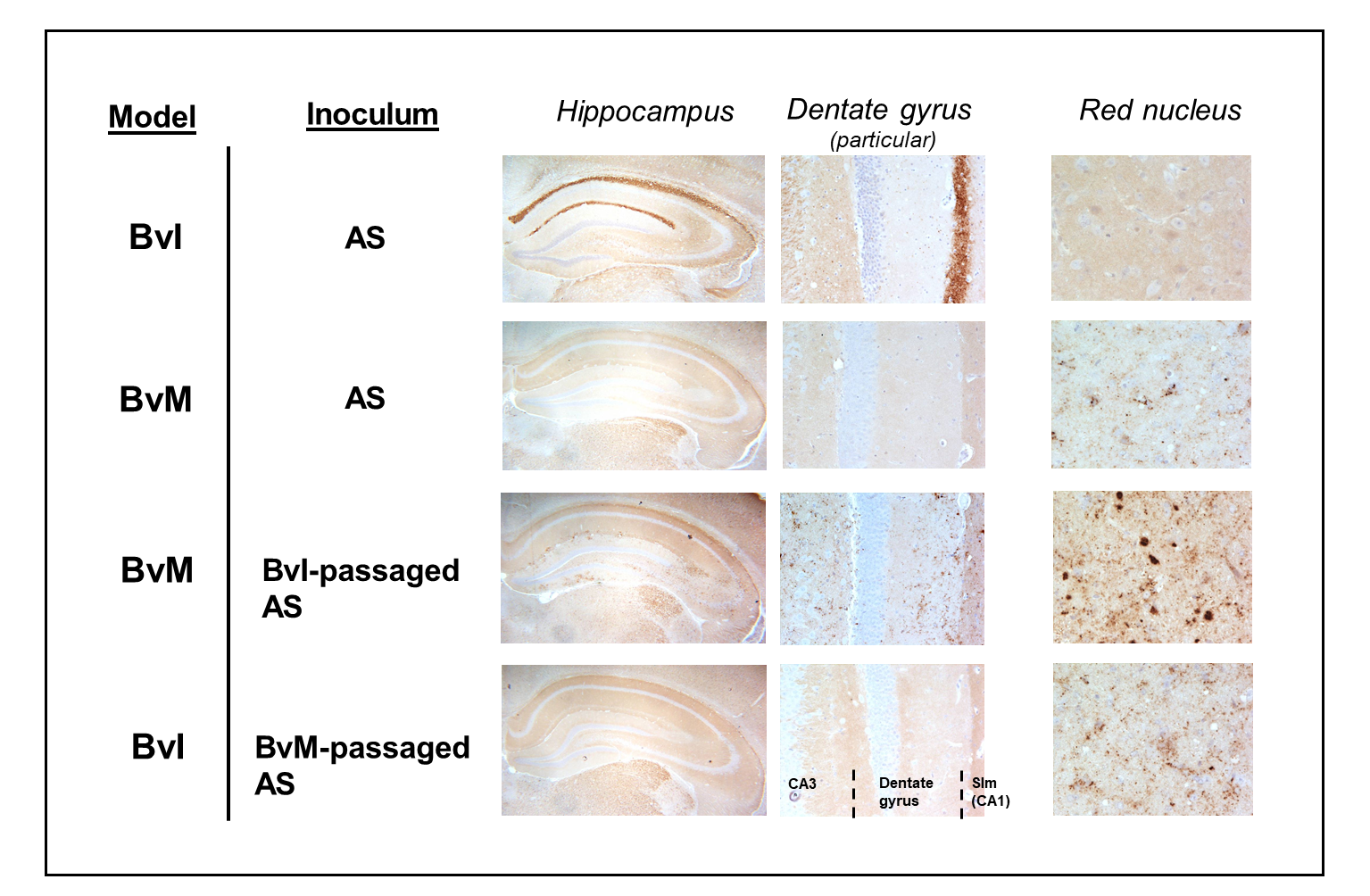

Supplement: S2 Fig — PrPSc deposition patterns observed in hippocampus and red nucleus of BvI and BvM inoculated with AS and with AS-passaged in BvM and BvI, as indicated. Note that AS in BvI showed a fine punctate pattern in the alveus and stratum lacunosum-moleculare (Slm) of CA1 and no labeling in the red nucleus, while AS in BvM was characterized by intraglial and intraneuronal PrPSc depositions in the red nucleus, accompanied by diffuse and punctate immunolabeling in the neuropil. PrPSc deposits in BvM inoculated with BvI-passaged AS were mainly intraglial and intraneuronal in the hippocampus and red nucleus, accompanied by punctate, granular and plaque-like deposits in the red nucleus. Finally, PrPSc deposits in BvI inoculated with BvM-passaged AS overlapped with those observed in BvM inoculated with AS, mainly characterized by by intraglial and intraneuronal PrPSc depositions. (TIF) [file ppat.1010646.s002.tif]

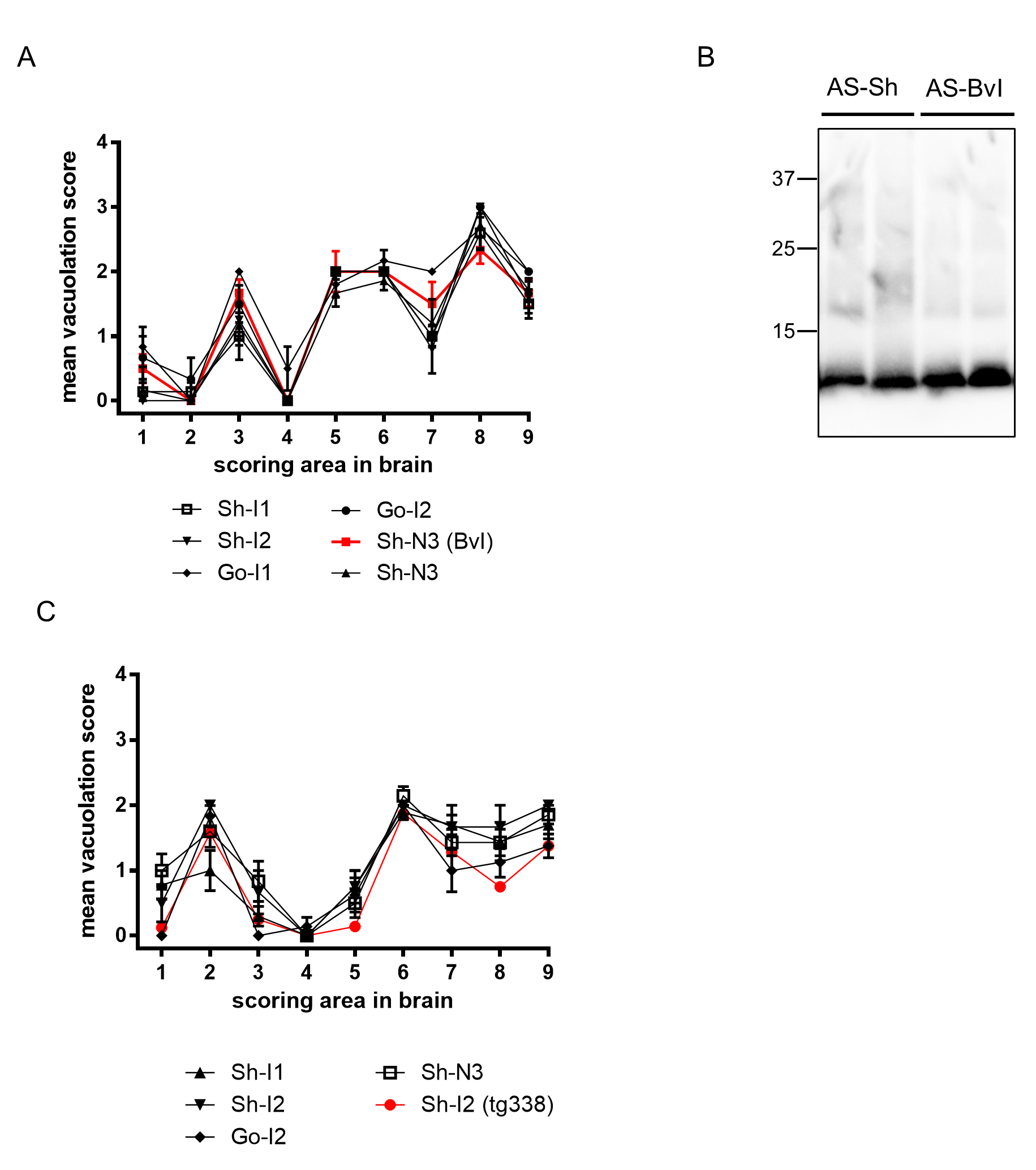

Supplement: S3 Fig — (A) Lesion profiles in groups of tg338 mice infected with AS natural isolates and BvI-passaged AS (Sh-N3 BvI). (B) Representative western blot showing PrPres from brains of tg338 infected with AS from sheep (AS-Sh) or with BvI-adapted AS (AS-BvI). (C) Lesion profiles in groups of BvI infected with AS natural isolates or with tg338-adapted AS (Sh-I2 tg338). (TIF) [file ppat.1010646.s003.tif]

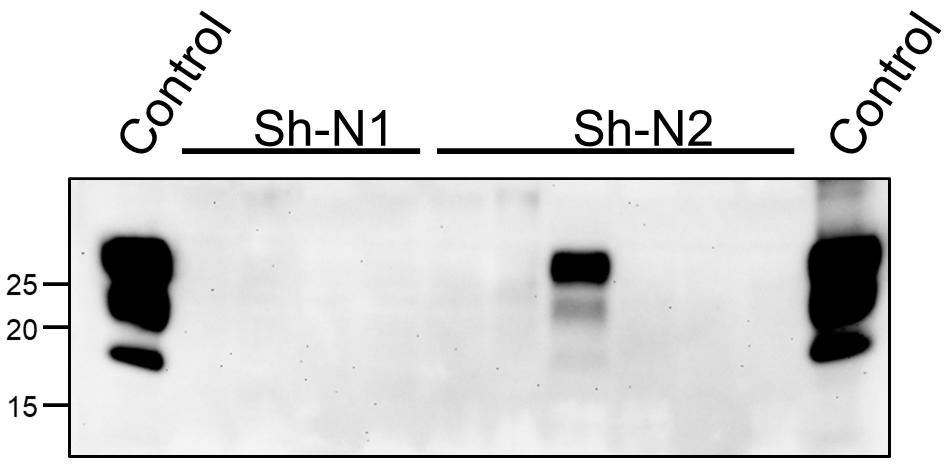

Supplement: S4 Fig — Representative western blot showing PK-resistant PrPSc from brains of BvM infected with AS isolates Sh-N1 and Sh-N2. As with the others AS isolates, most BvM were negative. The positive BvM shown is one of the two only BvM, both inoculated with Sh-N2, resulting in positive transmission. Note that PrPSc in BvM inoculated with AS is characterized by PrPres 27–30, similar to that in BvM inoculated with CS (control lanes) and different from AS in small ruminants and BvI. Membrane was probed with SAF84 mAb. The positions of MW markers are indicated on the left of the blot (in kilodaltons). (TIF) [file ppat.1010646.s004.tif]

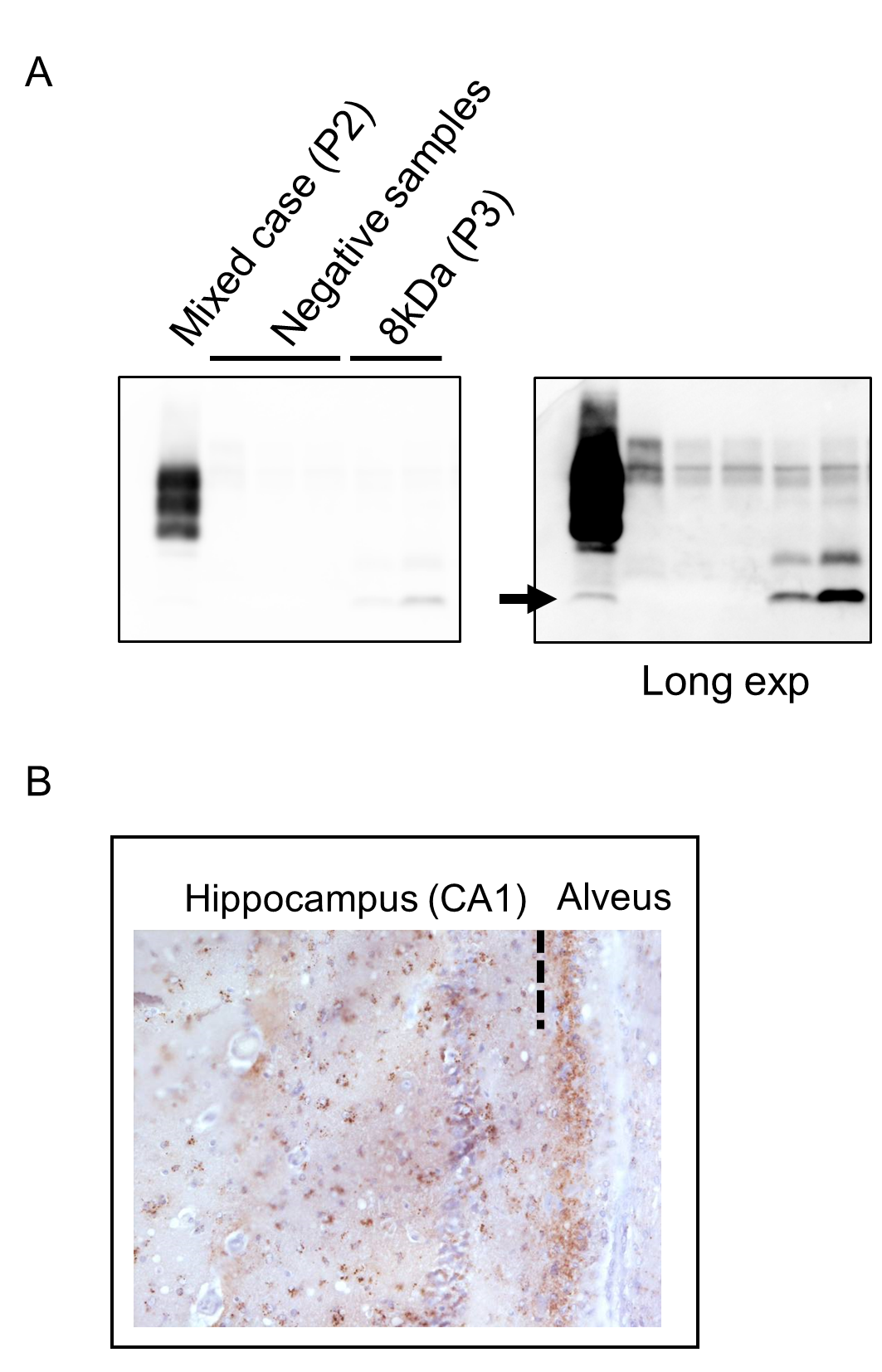

Supplement: S5 Fig — (A) Western blot of PrPres from the brain of individual BvI inoculated with Sh-N3 (second passage), Sh-N3 (third passage) or heathy controls, as indicated. The BvI from the second passage of Sh-N3 shows the classical 27–30 PrPres accompanied by small amount of the 8 kDa PrPres (indicated with the arrow in long exposure blot on the right). Membrane was probed with 12B2 mAb. (B) Immunohistochemical analysis of the mixed case highlights the simultaneous presence of intracellular PrPSc deposits in CA1, characteristic of BvM inoculated with AS, and fine punctate deposits in the alveus, characteristic of BvI inoculated with AS. (TIF) [file ppat.1010646.s005.tif]
